# Canned Fruits

**Published:** 1871-10

**Authors:** 


### CANNED FRUITS,

And vegetables, and meats, are now becoming of universal use. If well put up, a common tin can will keep its contents in a perfect state of preservation for any needed time. But by carelessness or want of skill the work of canning, especially in large establishments, is imperfectly done, with the result that the contents become sour, the metal decomposes, and poisonous combinations often take place; hence glass jars should always be preferred, — they only are safe. Perhaps after all, for domestic purposes, there is nothing better for "canning" fruit or other articles, than the old practice of fifty years ago: put the material in a common bottle, cork it well, turn the bottle upside down and bury it in the earth, or cover over with earth, two feet deep, to keep it out of the reach of frost. So great is the demand for canned food of various kinds, that it has been found necessary to devise some more rapid means of attaching labels to the cans, as it keeps one man very busy to label eight hundred in a day; but a man in Brooklyn has invented a machine which labels nine thousand cans in a day, causing a saving of ten cents a gross, which is quite an item when it is considered that one establishment in Baltimore packs up, in one year, forty million cans of oysters.

